# Identifying dyslexia in school pupils from eye movement and demographic data using artificial intelligence

**DOI:** 10.1371/journal.pone.0292047

**Published:** 2023-11-22

**Authors:** Soroosh Shalileh, Dmitry Ignatov, Anastasiya Lopukhina, Olga Dragoy

**Affiliations:** 1 Center for Language and Brain, HSE University, Moscow, Russia; 2 School of Data Analysis and Artificial Intelligence, Faculty of Computer Science, Moscow, Russia; 3 Rastle lab, Royal Holloway, University of London, London, United Kingdom; 4 Institute of Linguistics, Russian Academy of Sciences, Moscow, Russia; Jordan University of Science and Technology, JORDAN

## Abstract

This paper represents our research results in the pursuit of the following objectives: (i) to introduce a novel multi-sources data set to tackle the shortcomings of the previous data sets, (ii) to propose a robust artificial intelligence-based solution to identify dyslexia in primary school pupils, (iii) to investigate our psycholinguistic knowledge by studying the importance of the features in identifying dyslexia by our best AI model. In order to achieve the first objective, we collected and annotated a new set of eye-movement-during-reading data. Furthermore, we collected demographic data, including the measure of non-verbal intelligence, to form our three data sources. Our data set is the largest eye-movement data set globally. Unlike the previously introduced binary-class data sets, it contains (A) three class labels and (B) reading speed. Concerning the second objective, we formulated the task of dyslexia prediction as regression and classification problems and scrutinized the performance of 12 classifications and eight regressions approaches. We exploited the Bayesian optimization method to fine-tune the hyperparameters of the models: and reported the average and the standard deviation of our evaluation metrics in a stratified ten-fold cross-validation. Our studies showed that multi-layer perceptron, random forest, gradient boosting, and k-nearest neighbor form the group having the most acceptable results. Moreover, we showed that although separately using each data source did not lead to accurate results, their combination led to a reliable solution. We also determined the importance of the features of our best classifier: our findings showed that the IQ, gender, and age are the top three important features; we also showed that fixation along the y-axis is more important than other fixation data. Dyslexia detection, eye fixation, eye movement, demographic, classification, regression, artificial intelligence.

## Introduction: Background, previous work, and motivation

### Background and previous work

Developmental dyslexia is a learning disorder characterized by specific reading impairment, despite normal intelligence and oral language skills [1]. Children with dyslexia suffer from slow and effortful reading and impaired word recognition; hence, text comprehension, the ultimate objective of reading is unachievable, which negatively affects educational success, mental health, and social integration [2, 3]. Therefore, identifying dyslexia at an early stage is of significant importance for taking appropriate action [4–6].

Traditionally, dyslexia is identified during a formal assessment which involves a set of language and cognitive tasks, tapping into phonological and visual-spatial processing, reading of words, non-words, and texts, spelling abilities, etc [7]. Such assessment batteries require a trained specialist (usually a psychologist or other learning specialist), are time-consuming, and require overt children’s responses during some rather toxic behavior (e.g., reading of non-words). Thus, they are hardly suitable for screening, which is in great demand to decrease the age of dyslexia identification. Recently, a new set of automatic dyslexia detection solutions has emerged; these methods are based on Artificial Intelligence (AI) algorithms that have been applied to various data sources, ranging from eye-tracking to neuroimaging data.

According to [8], the latest and most comprehensive review of the application of AI to identify dyslexia, MRI, fMRI, face video or image, reading test errors, test scores, EEG, and eye tracking are the seven data types used to train AI algorithms. To the best of our knowledge, considering the number of unique data sets, eye-tracking-based (seven data sets, including the current research), EEG (six data sets), and MRI (five data sets) are the top three frequently used data types.

Considering AI methods to identify dyslexia, the author of [9] concisely reviewed 13 AI-based solutions to detect dyslexia up to the end of 2019. In a more recent survey [8], 22 solutions up to the beginning of 2021, including the original 13, were comprehensively reviewed. According to [8], the support vector machines [10], artificial neural networks, and random forest [11], in descending order, are the three most commonly applied AI classification algorithms. Calculating the accuracy, precision, and recall in a 10-fold cross-validation procedure, is the most frequently applied evaluation framework.

The majority of the papers which have been published after the two earlier surveys also pursued similar frameworks. More precisely, El Hmimdi et al. (2021) [12] analyzed the raw eye-tracking data sets from [13, 14] and proposed a new set of eye descriptor parameters as the input features to their classical set of classification algorithms and obtained approximately 82% accuracy. Raatikainen et al. [15] introduced a new eye movement data set for Finnish natives. They exploited random forests to extract the most informative features and fed them to support vector machines to detect dyslexia. AlGhamdi 2022 [16] used a publicly available dataset [17], obtained from online gamified test results, and proposed a novel ensemble recommendation to detect dyslexia with nearly perfect classification accuracy, while Kaisar and Chowdhury (2022) [18], using the same data set, initially achieved lower accuracy and then systematically reviewed the impact of various oversampling methods and proposed a hybrid method, employing oversampling and ensemble learning, which then achieved higher accuracy, although not as high as AlGhamdi’s results.

The authors of [19] collected a hand-written-character data set from Chinese children and created a multi-level multidimensional model. Vajs et al. [20] applied VGG16 neural network [21] on a slightly different version of the data set proposed [22] and obtained 87% accuracy. Later, in [23], they proposed a new feature space and obtained ROC AUC equal to 0.96 for logistic regression. These authors validated the previous findings on two different data sets in [24].

Previous eye-tracking studies of reading Russian-speaking children with and without dyslexia are few in number and focused on comparing fixation durations, progressive saccades, and regressions in these two groups of participants ([25–27]). Their findings were consistent with the results of other studies in alphabetical languages. Namely, all three studies agreed that children with dyslexia produced longer fixations and were more sensitive to word length and frequency compared to typically-developing readers. Also, Parshina et al. (2022) applied the ScanPath method to investigate which global reading processes children in grades 1 through 5 with and without dyslexia adopted to read entire sentences. The authors identified five reading processes and concluded that children with dyslexia relied on the same processes that their typically developing peers but with a 3-year reading delay. Importantly, no previous studies of reading in Russian have ever aimed to classify readers with and without dyslexia based on their eye movements.

### Motivation and contribution

Although the majority of these solutions have obtained acceptable performances and they are remarkably faster than the traditional methods of dyslexia diagnostics, most of them suffer from several shortcomings. This study aimed to (i) address some of the shortcomings of previously developed solutions, (ii) propose an robust AI-based solution to detect dyslexia, and (iii) investigate the psycholinguistic knowledge with the performance of our best AI model. In order to elaborate on our objectives and contributions, first, we concisely review the shortcomings of the previous solutions. We categorize those shortcomings into (i) data-related and (ii) AI-related categories. Among the plausible data types, it is natural to use eye-movement data to analyze reading impairment like dyslexia, and we focus on this data type. We summarized the characteristics of the six previously introduced eye-movement data sets in Table 1.

**Table 1:** Chronologically ordered summary of eye-tracking data sets to study dyslexia from eye movement using AI.

Reference	Control Group Size	High Risk of Dyslexia Size	Low Risk of Dyslexia Size	Age Range	Target Values		Language
					Discrete	Continuous	
[28]	97	88	0	9-10	2	-	Swedish
[29]	32	37	0	8.5-12.5	2	-	Greek
[22]	18	18	0	8-12	2	2	Serbian
[15]	135	30	0	ave. 12.5	2	-	Finnish
[12]	41	46	0	12.3-18	2	-	French
[30]	49	48	0	11-55	2	-	Spanish
This work	213	72	22	6-14	3	1	Russian

Concerning the data-related issues, the following observations from Table 1 require extra attention: (i-a) the size of the data sets, (i-b) synthetically balanced data set—except for [15], (i-c) the characteristics of the target values, (i-d) the age range, and (i-e) being limited to a specific language. More precisely, regarding (i-a), it is well known that the larger the data size, the greater the power of an AI model to recognize patterns [31, 32]. Our data set is the largest data set of its type, and thus, should increase the power of the AI models. As for (i-b), although synthetically balancing data representations is a popular method for addressing class imbalance issues; to the best of our knowledge, there is no rigorous mathematical definition to decide which samples should be selected for further up/down-sampling. Current techniques may lead the model to assign more weights to some of the data points in the synthetically manipulated data, and there is no guarantee that the new data representation is aligned with the unknown, underlying real-world distribution. The findings of [33], a recent and comprehensive review on this subject, are partially aligned with our line of thought and confirmed our claims. Therefore, we increased the size of our data set and, to some extent, preserved the imbalanced data representation.

Concerning (i-c), instead of binary-class data, which consists of typically developing and dyslexic readers, we introduced three classes: 1) typically developing readers, 2) those at low risk of dyslexia, and 3) those at high risk of dyslexia; additionally, we introduced a contiguous target variable of reading speed, which is a direct measure of reading aloud. This setting enabled us to formulate the problem both as classification and regression tasks. We intended to create a margin between the two traditional classes by introducing the low-risk class. Regarding (i-d), our data set covers a broader age range, and thus, we expected to detect dyslexia at its earlier stages among school pupils. Therefore, our newly introduced data set can be considered our first contribution.

Our second main objective and contribution addresses the AI-related shortcomings of previous papers. To the best of our knowledge, this is the most comprehensive empirical research scrutinizing the performance of 12 classifications and eight regression approaches for identifying dyslexia with the help of AI. The entire AI methods under consideration, with the help of the Bayes optimization search method, have been fine-tuned, and the corresponding tuned values are reported accurately.

Our third objective and contribution is introducing the application of the Shapley additive explanation approach, to determine the importance of the AI methods to this area of research, in order to investigate our psycholinguistic knowledge with the performance of our best AI method.

### Data sets

A fraction of the current data set, that is, 144 participants’ data, was reported in [27]. In that paper, the authors analyzed the eye movements of typical readers vs. children with dyslexia using ScanPath [34] and clustering methods. The current study pursues different objectives and adds 163 new participants’ data. All data collection of the current study was approved by the HSE Committee on Interuniversity Surveys and Ethical Assessment of Empirical Research and conducted in accordance with the Declaration of Helsinki (World Medical Association, 2013). The participants were recruited between March 2020 and March 2022. Their parents signed an informed consent form before the study. The authors have access to the participants eye-tracking and behavioral data, their age, grade, gender, and identification number. They have no access to information that could identify individual participants.

The complete data set used in this study, as well as the Python code for applying all of the methods and the metrics under consideration, are made publicly available in the following GitHub repository: https://github.com/Sorooshi/DD.

### Apparatus and stimuli

The eye-tracking data set was collected under well-controlled experimental conditions. The participants’ eye movements were recorded with an Eyelink 1000 Plus or an Eyelink Portable Duo eye-trackers (SR Research, Canada) with a sample rate of 1000 Hz. The participants were seated 55 cm from the camera while their heads were fixed using a chin rest. Only the right eye movements were tracked [35]. Natural reading performance was measured: the participants silently read 30 different sentences from the child’s version of the Russian sentence corpus [36, 37]. The selected sentences were suitable for primary school children and had diverse grammatical structures typical for the readers. The sentences were demonstrated in a random order for each participant. Ten sentences were followed by a two-option comprehension question, to check for involvement in the task. The task lasted approximately 20 minutes.

All participants’ data, regardless of their accuracy in the comprehension questions, were included in the analysis. Using the EyeLink Data Viewer software 4.2.1 (Oakville, Ontario, Canada: SR Research Ltd.), we generated a fixation report (also referred to eye-fixation in this paper), a interest area report (also referred to IA or IA data in this paper) from the collected raw eye movements, refer to [38, 39] for more details.

The fixation, IA and the demographic information–including the measure of non-verbal intelligence (IQ)–formed the three sources of our introduced data set in this paper. We combined demographic data with the fixation and with IA reports to test the additive value of demographics with the eye-tracking data.

To obtain an independent, direct, and continuous measure of reading performance, we also tested each participant with the Standardized Assessment of Reading Skills (SARS) tool [40]. Children had to read aloud a text (“How I caught a crayfish”) of 227 words in print form as quickly and as accurately as possible. The number of words read accurately in the first minute was taken as a measure of an individual child’s reading fluency.

### Demographic data

Our data set includes 307 Russian-speaking primary school students from first to sixth grade. All children had various, but age-appropriate nonverbal intelligence, assessed with Ravens colored progressive matrices [41]. The participants’ parents reported no abnormal vision capabilities and no history of neurological or psychiatric disorders. They also confirmed that their children are monolingual.

Based on the SARS test [40] and recent normative cutoff levels obtained in [42], individual reading performance was annotated into three groups: 1) typically developing children (TD); 2) children at risk of developmental dyslexia (DR); 3) children with developmental dyslexia (DD). The TD group, which we occasionally refer to as typical readers in this paper, consists of 213 students, 100 girls, and 113 boys. The DR group consists of 22 students, seven girls, and 15 boys. The DD group consists of 72 students, 27 girls, and 45 boys. We summarized the characteristics of our data set in Table 2.

**Table 2:** Summary of the demographic data set. *N* represents the number of participants.

	TD	DR	DD
Grade 1	*N* = 51 (22 girls, Age = 7.3 ± 0.5)	*N* = 6 (4 girls, Age = 7 ± 0.6)	*N* = 8 (2 girls, Age = 7.2 ± 0.5)
Grade 2	*N* = 40 (24 girls, Age = 8.3 ± 0.5)	*N* = 7 (1 girl, Age = 8.6 ± 0.5)	*N* = 10 (2 girls, Age = 8.4 ± 0.8)
Grade 3	*N* = 37 (19 girls, Age = 9.3 ± 0.5)	*N* = 1 (1 girl, Age = 9)	*N* = 20 (12 girls, Age = 9.3 ± 0.6)
Grade 4	*N* = 39 (18 girls, Age = 10.2 ± 0.5)	*N* = 2 (0 girls, Age = 10. ± 0.)	*N* = 28, (9 girls, Age = 10.2 ± 0.6)
Grade 5	*N* = 30 (12 girls, Age = 11.2 ± 0.8)	*N* = 2 (0 girls, Age = 11.5 ± 0.7)	*N* = 6 (2 girls, Age = 11.2 ± 0.4)
Grade 6	*N* = 16 (5 girls, Age = 12.1 ± 0.6)	*N* = 4 (1 girl, Age 11.7 = ± 0.5)	NA NA
Total	*N* = 213 (100 girls 113 boys)	*N* = 22 (7 girls 15 boys)	*N* = 72 (27 girls 45 boys)

The DR group refers to those students whose reading performance based on the SARS was between 1 and 1.5 standard deviation (SD) lower than the population average. This group consists of 22 students, seven girls, and 15 boys. The last group, DD, consists of students whose reading speed was lower than 1.5 SD of the populations average performance. This group consists of 72 students, 27 girls, and 45 boys. The borders between the groups were based on the SARS test guidelines.

### Eye-fixation data

In the fixation report, each row represents a fixation event arranged in the order of fixations in each sentence (for each participant). It includes information about the duration of the current fixation (*FIX*_*DURATION*) in milliseconds and the *x* and *y* fixation coordinates, *FIX*_*X* and *FIX*_*Y*, respectively.

### Interest area data

In the interest area report (IA), each row contains information about eye-movement events for each interest area (word) in each sentence (for each participant). The eye-movement events that we analyzed are as follows:

- *FIRST*_*FIXATION*_*DURATION*: the duration of the first fixation on a word;
- *FIRST*_*RUN*_*TOTAL*_*READING*_*TIME*: the sum of all fixations’ duration on a word during first-pass reading;
- *REGRESSION*_*PATH*_*DURATION*: the sum of all fixations’ duration on a word from the first fixation during first-pass reading until the eyes move to the right, including time spent re-reading;
- *TOTAL*_*READING*_*TIME*: the sum of all fixations duration on a word;
- *FIXATION*_*COUNT*: the total number of fixations on a word;
- *SKIP*: the probability of skipping a word;
- *FIRST*_*SACCADE*_*AMPLITUDE*: amplitude (in the degree of visual angle) of the first saccade to a word;
- *FIRST*_*FIXATION*_*X*: the *x* coordinate of the first fixation event on a word;
- *FIRST*_*FIXATION*_*Y*: the *x* coordinate of the first fixation event on a word;
- *REGRESSION*_*IN*: the probability of a backward saccade (regression) to a word;
- *REGRESSION*_*OUT*: the probability of regression from a word during first-pass reading;
- *REGRESSION*_*OUT*_*FULL*: the overall probability of regression from a word.

### Experiments setting

#### Preprocessing techniques

If the data set contains a categorical feature, we converted it to its one-hot encoded version. After such a conversion, if needed, all the data sets and their corresponding independent variables were standardized using the Min-Max technique, that is, each feature of a data point is subtracted from the corresponding minimum value and then divided by its range. More formally, if $D=\{d_{iv}{\}}_{i=1}^{N}$ for *v* = 1, …, *V*, where *V* is the number of features, it represents our data sets consisting of *N* data points; and *d*_*va*_ and *d*_*vb*_ denote the maximum and minimum of feature *v*. This techniques standardizes the data point $\hat{d_{iv}}=\frac{d_{iv}-d_{vb}}{d_{va}-d_{vb}}$ s.t. $\hat{d_{iv}}\in [0,1]$.

#### Hyperparameter tuning strategy

Various methods have been proposed for tuning the hyperparameters of AI algorithms. Interested readers may refer to [43], a relatively recent survey, for more details and comparisons of different tuning methods. Relying on this survey, we also exploited the Bayesian optimization [44], BO, to fine-tune the hyperparameters of the algorithms under consideration.

BO considers the parameter tuning process as a function of all possible combinations of an algorithm’s parameters. First, it constructs a surrogate function, next it utilizes the so-called acquisition function to score and determine the next evaluation points, i.e. the next hyperparameter setting in the optimization loop. More rigorously, BO optimizes

$$\begin{array}{c} \theta^{*}=\underset{\theta }{\text{argmin}}f\left(\theta \right) \end{array}$$ (1)

where ***θ*** represents the parameters of the algorithm to be tuned.

In principle, for the given number of iterations *T*, BO consists of the following steps:

For *t* = 1 to *T*:

1. construct a probabilistic model of the objective function *f* over the set $\{\theta_{i},y_{i}=f(\theta_{i}){\}}_{i=1}^{t}$. Integrate all the possible true functions using a Gaussian process or random forest regression;
2. optimize the acquisition function *u* based on the posterior distribution for sampling the next point i.e. ***θ***_*t*+1_ = argmin_***θ***_ *u*(***θ***);
3. sample the next observation *y*_*t*+1_ at ***θ***_*t*+1_.

We used random forest [11] and expected improvement (EI) as our surrogate and acquisition functions respectively. EI is defined as:

$$\begin{array}{c} -u\left(\theta \right)=-E[f\left(\theta \right)-f\left(\theta_{t}^{+}\right)] \end{array}$$ (2)

where $\theta_{t}^{+}$ is the best-observed hyperparameter setting. In our computations, we use Scikit-optimize [45] and Keras Tuner [46] Python libraries to tune the hyperparameters. The algorithms’ search spaces and the corresponding tuned hyperparameters are explained in the next section.

#### Computational setting

**Fig 1:**
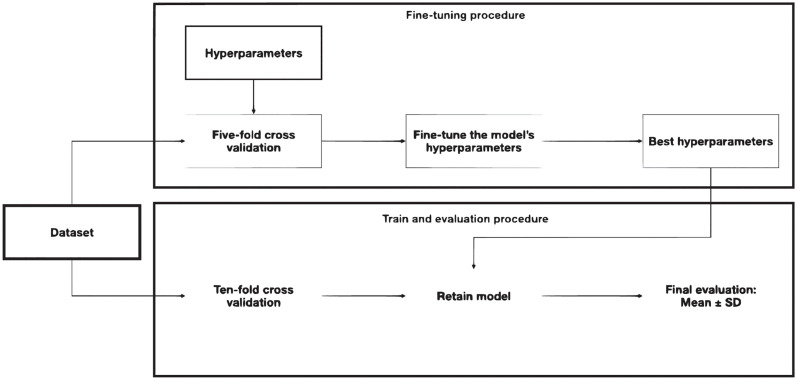
Adopted computation setting.

Our computations consisted of two components (i) fine-tuning the hyperparameters of the methods under consideration and (ii) a comprehensive evaluation of the fine-tuned methods. For adjusting the hyperparameters, we exploit BO and stratified k-fold cross-validation with *k* = 5. After fine-tuning the hyperparameters, we applied ten-fold cross-validation. At each fold, we trained an algorithm on the train split (90% of data) and evaluated it using the remaining unseen test data. Finally, we reported the average and standard deviation of evaluation metrics. Fig 1 demonstrates our computational setting.

#### Evaluation metrics

Let *y* be the set of true pairs of (datapoint, label). Similarly, let $\hat{y}$ be the set of predicted pairs of (datapoint, label). Let *L* and *S* be the sets of labels and datapoints, respectively. We defined *y*_*s*_ as the subset of *y* with sample *s*, that is, *y*_*s*_ ≔ {(*s*′, *l*) ∈ *y*|*s*′ = *s*}; and *y*_*l*_ as the subset of *y* with label *l*. Similarly, we defined ${\hat{y}}_{s}$ and ${\hat{y}}_{l}$ as analogous subsets of $\hat{y}$. Let $P(A,B)=\frac{\left|A\cap B\right|}{\left|B\right|}$, $R(A,B)=\frac{\left|A\cap B\right|}{\left|A\right|}$, and $F_{\beta }(A,B)=(1+\beta^{2})\times \frac{P(A,B)\times R(A,B)}{\beta^{2}\times P(A,B)+R(A,B)}$ for some sets *A* and *B*. We utilized precision, recall, and *F*1-score (*β* = 1) to assess the performance of the classification algorithms:

$$\begin{array}{c} precision=\frac{1}{\sum_{l\in L}\left|y_{l}\right|}\sum_{l\in L}\left|y_{l}\right|P\left(y_{l},\hat{y_{l}}\right), \end{array}$$ (3)

$$\begin{array}{c} recall=\frac{1}{\sum_{l\in L}\left|y_{l}\right|}\sum_{l\in L}\left|y_{l}\right|R\left(y_{l},\hat{y_{l}}\right), \end{array}$$ (4)

$$\begin{array}{c} F1\text{-score}=\frac{1}{\sum_{l\in L}\left|y_{l}\right|}\sum_{l\in L}\left|y_{l}\right|F\left(y_{l},\hat{y_{l}}\right). \end{array}$$ (5)

We also used the weighted average of the area under the receiver operating characteristic curve (ROC AUC) in a one-versus-rest manner to summarize the classifier’s performance at different discrimination thresholds for all classes. Noteworthy to add that all of the metrics under consideration are ∈ [0, 1], and the closer to unity, the better the performance of the model.

## Methods under consideration

One of the central objectives of this research was to conduct a comprehensive set of experiments to empirically scrutinize the performance of various AI methods for finding a robust AI-based solution to detect dyslexia. To this end, we studied the performance of four families of models: (A) artificial neural networks: multi-layer perceptron and convolutional neural network; (B) non-parametric: random forest, AdaBoost, Gradient Boosting, k-nearest neighbor, and support vector machines; (C) linear: linear regression and logistic regression; (D) Bayesian: Gaussian, multinomial, and complement naive Bayes. The multinomial and complement naive Bayes models did not obtain satisfactory results; therefore, we excluded them from the paper. Although we obtained similar results for both classification and regression tasks, for brevity, we only focused on explaining the classification tasks. The rest of this section describes the principles of the models mentioned above.

### Artificial neural networks

In our experiments, we exploited two methods of this family (1) the fully connected multi-layer perceptron (MLP) and (2) the convolutional neural network (CNN). The motivation for choosing them is merely due to their successful history.

#### Notation

Let x∈X and y∈Y, represent the data points and the target values, respectively. The goal is to learn a conditional probability distribution *p*(**y**|**x**, ***θ***) from training data, $D=\{(x_{i},y_{i}){\}}_{i=1}^{N}$, where *N* is the number of training cases, and ***θ*** represents the parameters of the model to be estimated.

#### Multi-layer perceptron

In principle, MLP adjusts the weights **W**_*ℓ*_ and the biases **b**_*ℓ*_ (for *ℓ* = 1, …, *L*) of the composition of *L* hidden layers to derive the distribution of a mapping function between the input data points **x** and target variables **y**, i.e. *p*(**y**|**x**; ***θ***), where ***θ*** = (**W**_1_, **b**_1_, …, **W**_*L*_, **b**_*L*_). In other words, let us denote the hidden units at layer *ℓ* with **z**_*ℓ*_ and the element-wise (non-)linear activation function with ψ:ℝ→ℝ, thus:

$$\begin{array}{c} z_{\ell }=f_{\ell }\left(z_{\ell -1}\right)=\psi_{\ell }\left(W_{\ell }z_{\ell -1}+b_{\ell }\right), \end{array}$$ (6)

Consequently, we can show the composition of all layers as:

$$\begin{array}{c} f\left(x;\theta \right)=f_{L}\left(f_{L-1}\left(\ldots .\left(f_{1}\left(x\right)\right)\ldots \right)\right). \end{array}$$ (7)

where, by convention, **z**_1_ = **x**.

At each layer of this composition, the gradients are computed w.r.t to their parameters using the chain rule, and then, those gradients (or higher order derivatives) are passed to an optimizer to adjust the parameters. Refer to chapter 13 of [47] for more details about MLP and chapters five and six of [48] about the popular optimization algorithms. The main hyperparameters of MLP are (i) the number of neurons, (ii) the number of hidden layers, (iii) the learning rate, (iv) activation functions, (v) the number of epochs, and (vi) the optimization algorithm. In this study, due to the limited size of the data set and to avoid overfitting, we limited ourselves to shallow networks and only used one hidden layer, and we fixed the batch size to 32. Table 3 shows the domain of the parameters and the corresponding tuned values.

**Table 3:** Multi-Layer Perceptron (MLP) and convolutional neural network methods: Hyperparameters’ domain and the corresponding tuned values at the data sets under consideration. The *N*_*n*_, *N*_*e*_, *lr*, in respect, represents the number of neurons of the hidden layer, the number of epochs, and the learning rate. The drop shows the maximum dropout in the max pooling layer.

method / data set	parameters					
	*N*_*n*_	*N*_*e*_	*lr*	activation	optimiser	Drop
MLP	{2, 3, …, 200}	[10, 50000]	[1e-6, 1e-2]	{Identity, Logistic, Tanh, ReLu}	{LBFGS, SGD, ADAM},	[0.1, 0.8]
MLP at Demo	173	31270	-	Identity	LBFGS	-
MLP at Fixation	190	58325	-	ReLu	LBFGS	-
CNN at Fixation	192	100	0.0001	ReLu	ADAM	0.1
MLP at Demo-Fixation	158	49150	-	Tanh	LBFGS	-
CNN at Demo-Fixation	192	200	0.0001	ReLu	ADAM	0.1
MLP at IA	34	66253	-	Logistic	LBFGS	
MLP at Demo-IA	50	13953	-	Logistic	LBFGS	

#### Convolutional neural networks

The core operation of an MLP hidden layer is to calculate the activation values **z** = *ψ*(**W** **x**), where **x** is the input to a layer, **W** are the weights, and *ψ*(.) is the activation function. Therefore, the *j*th component of the hidden layer has the value $z_{j}=\psi (w_{j}^{Τ}x)$. This inner product operation is equivalent to comparing the input **x** to a parameter **w**_*j*_. Due to non-shared weights across the location, it is not hard to show that this operation is not translation invariant.

Convolutional layers were proposed to tackle this issue. Although the name implies that convolution should be the core operation, when the weight vector is symmetric, which is often the case, convolution and cross-correlation are identical. Since the cross-correlation has fewer implementation difficulties, it is implemented more frequently. The cross-correlation between the weight vector **w** and input vector **x** is:

$$\begin{array}{c} \left[w*x\right]\left(i\right)=\sum_{u=0}^{K}w_{u}x_{i+u}, \end{array}$$ (8)

where *K* is the size of the kernel or filter and *x*_*i*_ represents the *i*th element of vector **x**.

It is not hard to show that this operation acts as a feature detector with an equivariance property–preserving information about the location of the input features. However, this property is not always desirable, and max pooling solves this problem by selecting the maximum value of its input within a predefined window. Max pooling operation or other options, like average pooling, form the so-called pooling layer. The composition of a convolutional layer and a pooling layer forms the cornerstone of a CNN. A generic and shallow architecture of a CNN usually consists of stacking a couple of pairs of convolutional-pooling layers, followed by a flattening layer and a shallow fully-connected MLP on top of it. The learning process is similar to MLP. For more details, refer to chapter 14 of [47].

The crucial hyperparameters of a CNN are: (i) the number of pairs of convolutional and pooling layers, (ii) the filter size, (iii) the number of filters, (iv) the number of hidden layers of MLP, (v) the number of neurons in MLP, (vi) the learning rate, (vii) the activation functions, (viii) the pooling size, (ix) the number of epochs. For the same reason mentioned about MLP, we limited ourselves to shallow networks and only used one hidden layer for MLP and two pairs of convolutional-pooling layers. We fixed the batch size at 32. Our preliminary experiments showed that fixing the filter size to three and choosing 32 filters led to the best performance of the CNN. The rest of the hyperparameters are tuned using BO. Table 3 shows the domain of the parameters and the corresponding tuned values.

From the reported tuned hyperparameters of Table 3 and considering the reported results of the next section, we may conclude that LBFGS [49] is the right optimiser for our problem using MLP. The difference between the number of epochs required to train MLP and CNN may require additional investigation. More interestingly, although the ReLu activation function is the most selected option, the best result, as reported in the next section, is obtained by MLP at the demo-fixation data set with the Tanh activation function.

#### Fusing CNN and MLP

**Fig 2:**
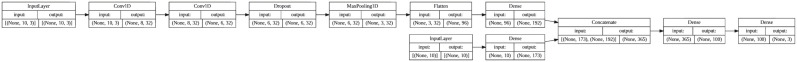
Fused CNN-MLP: Fusing the fine-tuned architectures of MLP at demographic data and CNN at fixation data to classify dyslexia from their combination.

As reported in the next section, MLP obtained the best ROC-AUC for identifying dyslexia from demographic data (see Table 8, and CNN performed the best at fixation-only data (see Table 11. Hence it is natural to combine these two models with their winning network architectures to identify dyslexia from the combination of fixation and demographic data. We named this model fused CNN-MLP and show the network architectures in Fig 2.

Recalling that the idea behind fusing CNN and MLP was to exploit the best of each model with their fined-tuned hyperparameters, thus, except for (A) the number of epochs, which we greedily searched for its best value between 1, 2000 epochs and ten epochs led to the best results, and (B) utilizing the ADAM optimizer with learning rate equal to 0.0001; the rest of hyperparameters and network architecture were identical to what reported in Table 3 and showed in Fig 2.

### Non-parametric models

#### Ensemble learning

A decision tree (DT) is a hierarchical tree structure that consists of a root node, internal nodes, and leaf nodes. The root node represents the entire data set and has no incoming edges. The leaf nodes represent all possible outcomes of the data set. DT aims to produce as pure leaf nodes as possible, i.e. in classification problems, the purity can be measured using entropy such that the purest leaf node will have an entropy equal or close to zero. To this end, DT recursively and greedily searches over the combination of all features and their values to find the best splitting point, i.e., the internal node which maximizes the information gain; the recursion terminates when a stopping condition is satisfied. For more details refer to [47].

DTs have several advantages, including being easy to interpret, fast to fit, and relatively robust to outliers. However, they are prone to overfitting, and they are high-variance estimators. Pre-pruning and post-pruning, i.e. controlling the tree’s depth and width, are popular techniques to prevent overfitting. However, reducing variance is more involved. One way is to use an ensemble of trees, for instance, random forests, RF, [11]. RF first builds various bootstrap samples from the training set and fits an unpruned learner, a decision tree, on each of the samples, and finally aggregates the predictions by voting. The generic model of an ensemble of *M* trees has the following form:

$$\begin{array}{c} t\left(y|x\right)=\frac{1}{M}\sum_{m\in M}\alpha_{m}t_{m}\left(y|x\right), \end{array}$$ (9)

where *t*_*m*_ is the *m*-th tree, *α*_*m*_ is the corresponding weight. We can think of this as an additive linear model with adaptive basis functions, and thus we can employ the steepest descent with line search and Boosting algorithms. AdaBoost, AB, [50] and Gradient descent Boosting, GB, [51] are based on this idea. They sequentially fit a weak learner, and at each sequence, they weigh the data to bias the next learner for correcting the mistakes of the current estimator and finally aggregate the weak learners to build a strong learner. In our opinion, AB can be considered as a specific case of GB with exponential loss; however, at each sequence, AB uses the learning rate to assign more weight to the prediction errors while GB shrinks the contribution of each tree to avoid overfitting.

The number of estimators, the minimum number of samples required to split an internal node, the minimum number of samples required to be at a leaf node, and the learning rate (in AB and GB) can be considered their most important hyperparameters. Table 4 provides more details on the hyperparameters and the tuned values of three ensemble learning methods.

**Table 4:** Random Forest (RF), Gradient Boosting (GB), and AdaBoost (AB) methods: Hyperparameters’ domain and the corresponding tuned values at the data sets under consideration. The *N*_*e*_, *M*_*ss*_, *M*_*sl*_, *lr*, in respect, represents the number of estimators, minimum number of samples per split, minimum number of samples per leaf, and learning rate.

method / data set	parameters			
	*N*_*e*_	*M*_*ss*_	*M*_*sl*_	*lr*
AB	{10, 11, …, 10000}	-	-	[1e-3, 5e-1]
RF	{10, 11, …, 10000}	{2, 3, …, 10}	{1, 2, …, 10}	-
GB	{10, 11, …, 10000}	{2, 3, …, 10}	{1, 2, …, 10}	[1e-3, 5e-1]
AB at Demo	545	-	-	0.017
RF at Demo	6197	5	9	-
GB at Demo	257	4	1	0.005
AB at Fixation	415	-	-	0.169
RF at Fixation	2726	2	10	-
GB at Fixation	3380	3	6	0.007
AB at IA	4736	-	-	0.087
RF at IA	1980	4	3	-
GB at IA	165	2	10	0.160
AB at Demo-Fixation	309	-	-	0.215
RF at Demo-Fixation	9923	9	1	-
GB at Demo-Fixation	2674	9	3	0.282
AB at Demo-IA	7133	-	-	0.019
RF at Demo-IA	163	2	1	-
GB at Demo-IA	971	7	3	0.299

Considering the results in the next section, although RF and GB obtained acceptable results, we cannot find any patterns in the tuned hyperparameter value, except considering this table as another piece of evidence confirming the so-called no-free-lunch theorem.

#### K-nearest neighbors

The k-nearest neighbors [52], (KNN) predicts the target value of an unseen data point **x** by deriving the distribution over the target values of its *K* nearest neighbors in the training set, i.e. $N_{K}(x,D)$. More precisely,

$$\begin{array}{c} p\left(y=c|x,D\right)=\frac{1}{K}\sum_{n\in N_{K}\left(x,D\right)}I\left(y_{n}=c\right), \end{array}$$ (10)

where I is an indicator function, returning one when the condition is satisfied and zero otherwise.

KNN has two major hyperparameters: (i) the number of nearest neighbors and (ii) the choice distance metric to define the neighborhood of **x**, i.e. *d*(**x**, **x**′). We used the Minkowski distance and treated its value of *P* as a hyperparameter. Table 5 provides more details on the hyperparameters and the corresponding tuned values.

**Table 5:** K-nearest neighbours regression (KNN) methods: Hyperparameters’ domain and the corresponding tuned values at the data sets under consideration. The *K*, *P*, in respect, represents the number of nearest neighbours and the value of *P* in the Minkowski distance metric.

method / data set	parameters	
	*K*	*P*
KNN	{1, 2, …, 10}	[1, 5]
KNN at Demo	10	1.976
KNN at Fixation	10	1.214
KNN at IA	10	1.544
KNN at Demo-Fixation	9	1.042
KNN at Demo-IA	5	1.013

In our opinion, the tuned values of *P* at the Minkowski distance, which is always greater than one and less than two, may require scrutinizing to justify the underlying reasons why the Minkowski distance works better in this range.

#### Support vector machines

Support vector machines (SVM) maximize the margins between the hyperplane and the support vectors. There have been various proposed kernel extensions of SVM see [53, 54]. The hyperparameters of these extensions consist of: (i) kernel type, i.e., linear, polynomial, RBF, or sigmoid; (ii) the value of the regularization term *c*, (iii) kernel coefficient *γ* for the case of using non-linear kernels; (iv) degree of the polynomial kernel, (v) the epsilon-tube value *ϵ* (within which no penalty is associated in the training loss function with points predicted within a distance epsilon from the actual value). See Table 6 for more details on the hyperparameter domains and their tuned values.

**Table 6:** Support vector machine (SVM): Hyperparameters’ domain and the corresponding tuned values at the data sets under consideration. The *c*, *γ*, *ϵ*, in respect, represents the regularisation term, kernel coefficient, and epsilon-tube (applicable only to linear SVM) value.

method / data set	parameters				
	kernels	degree	*c*	*γ*	*ϵ*
SVM	{linear, poly, RBF, sigmoid}	{1, 2, 3}	[1, 4]	[-2.3, 0.7]	[-2.3, 0.7]
SVM at Demo	sigmoid	-	0.333	0.504	-
SVM at Fixation	poly	3	1.640	0.563	-
SVM at IA	rbf	1	3.652	1.933	-
SVM at Demo-Fixation	RBF	2	3.929	1.941	-
SVM at Demo-IA	poly	3	3.950	1.987	-

The kernel function maps a non-linear feature space of the training data into a linearly separable feature space. SVM performed best in the demo-fixation data set with RBF kernels with a degree equal to two and a regularization term equal to 3.93. And its best performance obtained at IA-demo with polynomial kernel and a regularization term equal to 3.95.

### Naive Bayes classifiers and logistic regression

The underlying assumption in naive Bayes classifiers is the conditional independence of the features, given the class label. Concretely, this assumption corresponds to the following class conditional density:

$$\begin{array}{c} p\left(x|y=c,\theta \right)=\prod_{v=1}^{V}p\left(x_{v}|y=c,\theta_{vc}\right), \end{array}$$ (11)

where ***θ***_*vc*_ are the parameters of the class conditional density of class *c* and feature *v*. Therefore, we can compute the posterior over the class labels as follows:

$$\begin{array}{c} p\left(y=c|x,\theta \right)=\frac{p\left(y=c|\pi \right)\prod_{v=1}^{V}p\left(x_{v}|y=c,\theta_{vc}\right)}{\sum_{c^{\prime }}p\left(y=c^{\prime }|\pi \right)\prod_{v=1}^{V}p\left(x_{v}|y=c^{\prime },\theta_{vc^{\prime }}\right)}, \end{array}$$ (12)

where *π*_*c*_ ∈ ***π*** is the prior probability of class *c*, and it is equal to the relative frequency of each class in the training set. Depending on the assumed distribution for *p*(*x*_*v*_|*y* = *c*, ***θ***_*vc*_), different versions of the naive base classifier have been proposed. In our experiments, multivariate Gaussian distribution led to the most satisfactory results among the members of this family, and thus we limited our report to it. To fit the model, first, we need to select the proper distribution, next by applying maximum likelihood estimation and gradient descent, we fit the model to the data. It is not hard to show that the optimal parameters for the multivariate Gaussian distribution are:

$$\begin{array}{c} {\hat{\mu }}_{vc}=\frac{1}{N_{c}}\sum_{n:y_{n}=c}x_{nv}, \end{array}$$ (13)

and

$$\begin{array}{c} {\hat{\sigma }}_{vc}^{2}=\frac{1}{N_{c}}\sum_{n:y_{n}=c}{\left(x_{nv}-{\hat{\mu }}_{vc}\right)}^{2}. \end{array}$$ (14)

While in naive Bayes classification we optimize the joint likelihood ∏_*n*_ *p*(*y*_*n*_, **x**_*n*_|***θ***), in logistic regression we optimize the conditional likelihood ∏_*n*_ *p*(*y*_*n*_|**x**_*n*_; ***θ***). Concretely, multinomial logistic regression has the following form:

$$\begin{array}{c} p\left(y_{n}|x_{n},\theta \right)=Cat\left(y|S\left(Wx+b\right)\right), \end{array}$$ (15)

where $x\in {\mathbb{R}}^{V}$ is the data point, *y* ∈ {1, …, *C*} is the class label, **W** is the *C* × *V* weight matrix, **b** is the *V*-dimensional bias vector, *S*() is the softmax function, and ***θ*** = (**W**, **b**) are the model parameters. For model fitting the procedure described earlier can be adopted. Table 7 shows the domains’ parameters of the logistic regression algorithm and its corresponding optimal parameters found by BO during the training process at different data sets.

**Table 7:** Logistic Regression (LR): Hyperparameters’ domain and the corresponding tuned values at the data sets under consideration. The *C*, *N*_*i*_, *l*_1_ ratio, in respect, represents the inverse of regularization strength, the number of iterations, and the Elastic-Net mixing parameter.

method / data set	parameters				
	intercept	*C*	*N*_*i*_	*l*_1_ ratio	penalty options
LR	{*False*, *True*}	[1e-1, 4]	{100, 101, …, 100000 }	[1e-1, 9e-1]	{none, *l*_1_, *l*_2_, Elastic Net}
LR at Demo	True	0.4078	29307	0.1445	l2
LR at Fixation	True	0.1302	27169	0.2779	none
LR Demo-Fixation	True	0.1035	85814	0.8695	l1
LR at IA	True	3.123	83030	0.663	l2
LR at Demo-IA	False	0.317	90860	0.611	none

### Determine the importance of feature

After finding the best plausible fine-tuned estimator, we determined the importance of the features. We applied the Shapley Additive exPlanation (SHAP) approach and its Python library [55]. SHAP connects optimal payoff allocation with local explanations using the Shapley values from cooperative game theory and their related extensions.

In the machine learning setting, each attribute (or feature) of a given dataset is considered a player. Such players can negotiate and form coalitions (subsets of attributes). In the exhaustive case, the importance of each attribute *a* for the classification of an object *x* is counted over all possible combinations of this attribute with subsets *S* of all the remaining attributes with respect to a chosen value function as follows [56]:

$$\begin{array}{c} \varphi_{a}\left(x\right)=\sum_{S\subseteq \{1,\ldots ,m\}\backslash \{a\}}\frac{|S|!(m-|S|-1)!}{m!}\left(v\left(x,S\cup \left\{a\right\}\right)-v\left(x,S\right)\right), \end{array}$$ (16)

where *m* is the total number of attributes, *v* is the chosen value function.

In the simplest case, as explained in [57], the function value *v* is binary, 1 for winning coalitions, and 0, otherwise. If the coalition *S* ∪ {*a*} is winning (e.g., if *x* is classified correctly), while *S* is not, the attribute *a* receives a non-zero importance value. However, for large sets of attributes, the direct approach is no longer applicable due to a combinatorial explosion in terms of the number of possible coalitions, and the value function is expressed in terms of the approximate expectation computed, e.g., via Monte-Carlo approach [56].

We exploit two tools of the SHAP library 1) the bar plot of the Mean Absolute SHAP (MAS) values per feature and 2) the beeswarm summary plot. The MAS, on average, quantifies the magnitude of each feature’s contribution toward the predicted class labels. The higher the MAS value of a feature, the higher its impact. The rows of these two plots represent the data set features ranked in descending order, top-to-bottom. In each row of the beeswarm summary plot, points are distributed horizontally according to their SHAP value; in places with a high density, SHAP values are stacked vertically. Investigating how the SHAP values are distributed demonstrates a feature’s influence on the predictions. The color bar corresponds to value of each feature of the data point on the graph. If the value of a feature for a particular instance is relatively high, it appears as a yellow dot; while relatively low variable values appear as blue dots. Examining the color distribution along the *x*-axis for each variable provides insights into the general relationship between a variable’s raw values and its SHAP values.

## Experiments

### Experimental results and analysis

Tables 8 to 10 represent the results of the methods under consideration using demographic, IA and fixation data respectively. In predicting dyslexia from demographic data, GB outperformed the competitors and can be considered a low-quality winner. Also, it was the winner in the IA competition with relatively better results. CNN was the best using the fixation data with relatively acceptable results.

**Table 8:** Classification on demographic data set: The average and standard deviation of evaluation metrics over 10 different data splits. The best results are bold-faced.

Methods	Metrics			
	Precision	Recall	*F*1-score	ROC-AUC
Logistic Regression	0.481 ± 0.019	0.691 ± 0.016	0.567 ± 0.018	0.619 ± 0.088
Gaussian Naive Bayes	0.065 ± 0.012	0.235 ± 0.029	0.100 ± 0.014	0.633 ± 0.061
Support Vector	0.482 ± 0.019	0.694 ± 0.014	0.569 ± 0.018	0.557 ± 0.102
K-Nearest Neighbour	0.543 ± 0.092	0.665 ± 0.051	0.577 ± 0.046	0.586 ± 0.106
Random Forest	0.480 ± 0.021	0.687 ± 0.028	0.565 ± 0.024	0.603 ± 0.103
Gradient Boosting	**0.547 ± 0.103**	**0.701 ± 0.033**	**0.595 ± 0.050**	0.558 ± 0.142
AdaBoost	0.523 ± 0.097	0.694 ± 0.031	0.582 ± 0.048	0.541 ± 0.109
Multi-Layer Perceptron	0.543 ± 0.094	0.684 ± 0.031	0.582 ± 0.031	**0.609 ± 0.071**

**Table 9:** Classification on IA report data set: The average and standard deviation of evaluation metrics over 10 different data splits. The best results are bold-faced.

Methods	Metrics			
	Precision	Recall	F1-score	ROC-AUC
Logistic Regression	0.656 ± 0.012	0.701 ± 0.003	0.619 ± 0.004	0.695 ± 0.007
Gaussian Naive Bayes	0.623 ± 0.007	0.668 ± 0.005	0.633 ± 0.005	0.661 ± 0.006
Support Vector	0.672 ± 0.031	0.702 ± 0.003	0.600 ± 0.003	0.679 ± 0.009
K-Nearest Neighbour	0.642 ± 0.009	0.698 ± 0.003	0.636 ± 0.004	0.664 ± 0.007
Random Forest	**0.705 ± 0.013**	0.720 ± 0.002	0.665 ± 0.003	0.727 ± 0.007
Gradient Boosting	0.696 ± 0.007	**0.723 ± 0.002**	**0.671 ± 0.003**	**0.738 ± 0.002**
AdaBoost	0.676 ± 0.013	0.715 ± 0.004	0.657 ± 0.005	0.709 ± 0.005
Multi-Layer Perceptron	0.693 ± 0.007	0.721 ± 0.003	0.671 ± 0.004	0.732 ± 0.007

**Table 10:** Classification on fixation report data set: The average and standard deviation of evaluation metrics over 10 different data splits. The best results are bold-faced.

Methods	Metrics			
	Precision	Recall	*F*1-score	ROC-AUC
Logistic Regression	0.499 ± 0.003	0.618 ± 0.001	0.499 ± 0.001	0.607 ± 0.002
Gaussian Naive Bayes	0.560 ± 0.005	0.618 ± 0.002	0.526 ± 0.003	0.606 ± 0.002
Support Vector	0.383 ± 0.000	0.619 ± 0.000	0.473 ± 0.000	0.577 ± 0.010
K-Nearest Neighbour	0.559 ± 0.003	0.605 ± 0.002	0.544 ± 0.002	0.599 ± 0.003
Random Forest	0.593 ± 0.004	0.626 ± 0.002	0.548 ± 0.002	0.637 ± 0.002
Gradient Boosting	0.602 ± 0.005	0.630 ± 0.002	0.545 ± 0.003	0.650 ± 0.004
AdaBoost	0.530 ± 0.003	0.626 ± 0.001	0.518 ± 0.001	0.628 ± 0.002
Multi-Layer Perceptron	0.600 ± 0.004	0.629 ± 0.001	0.541 ± 0.002	0.647 ± 0.003
Convolutional neural networks	**0.656 ± 0.077**	**0.673 ± 0.053**	**0.637 ± 0.056**	**0.758 ± 0.075**

Since the demographic data is not informative about a person’s reading ability, thus it is not a surprise that the results using fixation data are better than demographic. Moreover, no temporal or spatial relation is expected in demographic or IA data; therefore, applying CNN did not make sense. Comparing the best-obtained results over the three data sources, the winner of the IA dataset obtained a slightly better *F*1-score: while the winner of the fixation competition obtained a slightly better ROC AUC.

Table 11 represents the results using the combination of demographic and fixation report data sets. The combination of the two data sources significantly improved the algorithm’s performances, so that MLP obtained the best results with an acceptable *F*1-score = 0.912±0.002 and ROC-AUC = 0.983. RF obtained very similar results. The KNN and GB also performed acceptably with slightly worse results. The absence of a statistically significant auto-correlation in the demographic data may justify the obtained result by CNN. In our opinion, the difference between the required number of epochs to train CNN on the fixation data (100 epochs) and MLP on the demographic data (31,270 epochs) might justify the quality of the obtained results by the fused CNN-MLP.

**Table 11:** Classification on the combination of fixation report and demographic data sets: The average and standard deviation of evaluation metrics over 10 different data splits. The best results are bold-faced and the second ones are underlined.

Methods	Metrics			
	Precision	Recall	*F*1-score	ROC-AUC
Logistic Regression	0.573 ± 0.003	0.658 ± 0.002	0.599 ± 0.003	0.713 0.003
Gaussian Naive Bayes	0.724 ± 0.018	0.302 ± 0.001	0.162 ± 0.001	0.689 ± 0.003
Support Vector	0.807 ± 0.003	0.807 ± 0.003	0.802 ± 0.003	0.872 ± 0.002
K-Nearest Neighbour	0.903 ± 0.001	0.903 ± 0.001	0.903 ± 0.001	0.976 ± 0.001
Random Forest	0.911 ± 0.002	0.910 ± 0.002	0.911 ± 0.002	0.981 ± 0.001
Gradient Boosting	0.902 ± 0.002	0.901 ± 0.003	0.901 ± 0.003	0.978 ± 0.001
AdaBoost	0.669 ± 0.007	0.684 ± 0.002	0.642 ± 0.003	0.724 ± 0.002
Multi-Layer Perceptron	**0.913 ± 0.002**	**0.911 ± 0.002**	**0.912 ± 0.002**	**0.983 ± 0.000**
Convolutional neural networks	0.657 ± 0.097	0.649 ± 0.096	0.641 ± 0.096	0.713 ± 0.111
Fused CNN-MLP	0.685 ± 0.098	0.692 ± 0.094	0.675 ± 0.097	0.767 ± 0.098

Table 12 represents the results using the combination of IA and demographic data. We observed similar patterns and slightly better results. Although the IA report and its combination with demographic data led to slightly better results; however, since the fixation data is one of the purest reports one can obtain from the eye tracker and relying on the prior knowledge that the natural visual streams are modulated by fixations [58], in the remainder of this research we focused on the combination of fixation and demographic data.

**Table 12:** Classification on the combination of IA report and demographic data sets: The average and standard deviation of evaluation metrics over 10 different data splits. The best results are bold-faced.

Methods	Metrics			
	Precision	Recall	F1-score	ROC-AUC
Logistic Regression	0.722 ± 0.010	0.747 ± 0.003	0.702 ± 0.003	0.778 ± 0.005
Gaussian Naive Bayes	0.689 ± 0.008	0.356 ± 0.004	0.313 ± 0.006	0.733 ± 0.005
Support Vector	0.842 ± 0.006	0.846 ± 0.005	0.837 ± 0.006	0.858 ± 0.005
K-Nearest Neighbour	0.848 ± 0.004	0.852 ± 0.004	0.846 ± 0.005	0.920 ± 0.005
Random Forest	0.883 ± 0.004	0.881 ± 0.004	0.875 ± 0.004	0.957 ± 0.002
Gradient Boosting	0.914 ± 0.003	0.914 ± 0.003	0.913 ± 0.003	0.977 ± 0.001
AdaBoost	0.743 ± 0.008	0.771 ± 0.005	0.739 ± 0.006	0.761 ± 0.009
Multi-Layer Perceptron	**0.934 ± 0.005**	**0.934 ± 0.005**	**0.934 ± 0.005**	**0.986 ± 0.001**

**Fig 3:**
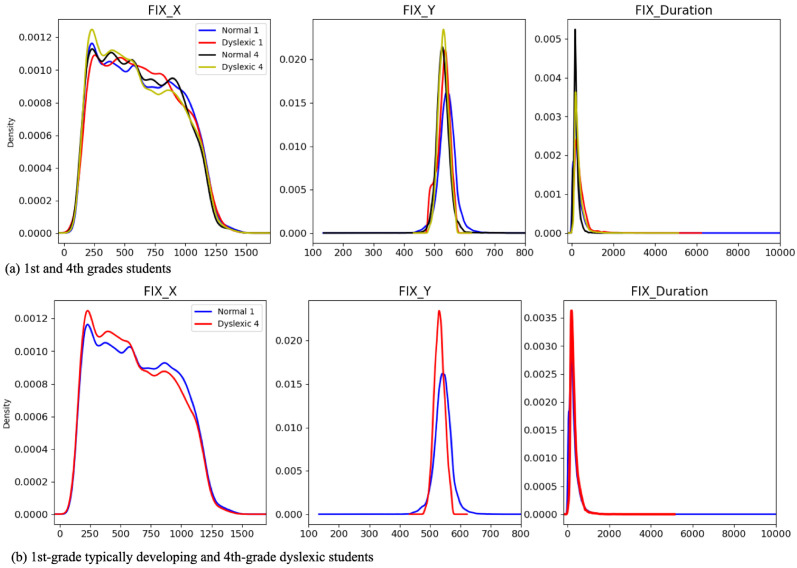
KDE of plots of fixation data: (a) both of the two grades under consideration, (b) the first-grade typically developing first-grade vs. dyslexics fourth-grade students.

We justify the improvement obtained from this combination(s) due to (1) large number theory, that is, combining the demographic and fixation (IA) data acts like a data augmentation technique which led to improvements in the performance of the AI models, and (2) the supplementary role of the demographic features for discriminating eye-fixation (IA) data of the three different classes. While (1) is quite well-known in the AI field; (2) aligns with our domain knowledge–the expected similarity between the eye movement of older students with dyslexia and younger non-dyslexics, as reported in [27]. As an additional assessment, we utilized the Gaussian kernel density estimation (KDE) with automatic bandwidth determination [59] to non-parametrically estimate and compare the probability density functions of non-dyslexic first-grade students with fourth-grade dyslexic students in our fixation report data set. Fig 3 demonstrates the similarities between the fourth-grade students with dyslexia and the first-grade students without dyslexia.

Last but not least, in each comparison, the proximity of the top three results and the fact that each algorithm has been tuned separately and then trained and evaluated on ten different disjoint train-test splits can be considered an acceptable evaluation policy to examine the generalizability power of the algorithms. In other words, the likelihood of the occurrence of overfitting (under-fitting) for two or more algorithms over ten different data splits is less than the case in which one simply applies only one algorithm.

### Feature importance

**Fig 4:**
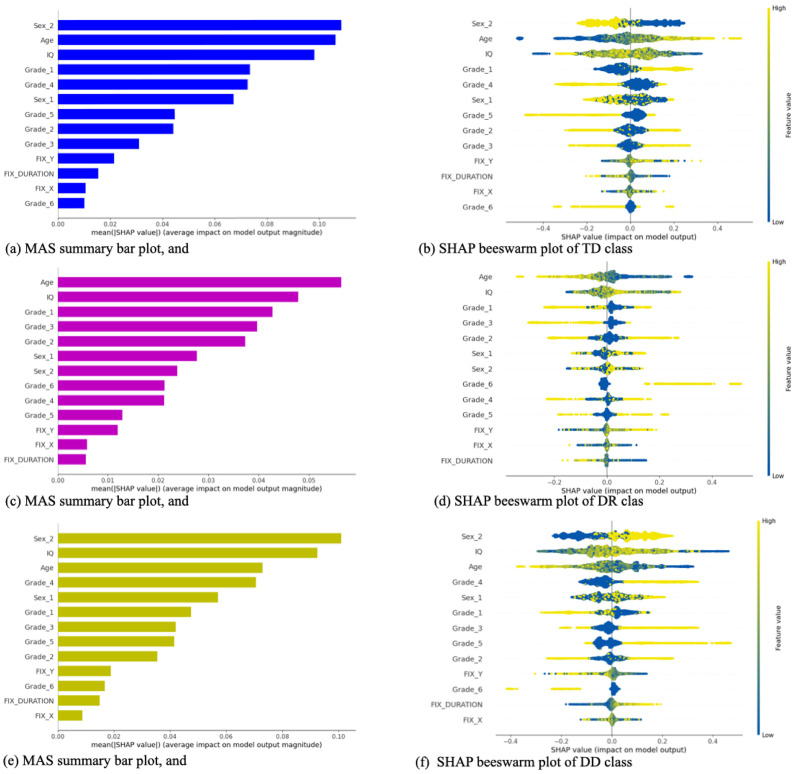
The first to third rows represent the accumulative MAS bar plots (on the left side) and the beeswarm plots (right side) of typically reading (TD), at the risk of dyslexia class (DR), and developmental dyslexia (DD) classes.

We scrutinized the importance of features on the MLP predictions, one of our best models, using the kernel explainer from the SHAP library, using the demographic-fixation data set. Due to the computational complexity of the SHAP approach, it was not feasible to use the entire train set as the background data, thus following the recommendation of the author of SHAP, first, we trained the MLP on the whole train split, and then passed the trained model with 500 randomly selected data points, from the train data split, as the background data to the kernel explainer, and we used the entire test split to determine the shape values. The results are illustrated in Fig 4. The left-hand side of this Fig. depicts the summary bar plot of the Mean Absolute SHAP (MAS) values of each feature per class, and its right-hand side depicts beeswarm summary plots of the TD, DR, and DD classes.

According to reported results, the demographic features, in total, formed 93.2% of MAS values, and the fixation data formed the remaining 6.8%. More precisely, considering the provided MAS summary bar subplots, the IQ, Age, and Sex_2, i.e., being male, with accumulative MAS values equal to 0.24, 0.23, and 0.23 respectively, were the three most important features. And among the six school grades, the first and the fourth grades (each with MAS = 0.16) were more important than the others. Considering the beeswarm summary plots (b, d, and f), we observed that being male was the most important feature in predicting TD and DD classes. Age was the most important feature in predicting DR and the second most important feature for identifying TD class. IQ was the second most important feature to predict DR and DD and was the third most important feature in predicting TD. Its two extremes had reverse impacts on the model’s predictions, especially in predicting TD and DD. It ought to emphasize that, in our opinion, the demographic features are more likely to be confounders than colliders. A deeper investigation of this subject is our future work agenda.

Regarding the fixation features, fixation along the y-axis, with the approximate MAS value of 0.052, had more impact than fixation duration with an approximate MAS value of 0.036 and fixation along the x-axis with MAS value of ≈0.025. We observed in our data set that students with dyslexia, on average, looked at lower positions on the screen while reading than typical readers. They had more frequent eye-movement leaps along the y-axis than typical readers. These two reasons justify why the model assigned a higher weight to this feature.

### Independent test results

Due to the real-world significance of the dyslexia screening problem and before launching a clinical trial of our proposed solution, we evaluated the performance of the tuned MLP classifier on an independent (and newly collected) test set using the combination of demographic and fixation data. This new test set consisted of nine typical readers (five girls) and seven students with dyslexia (four girls).

For a fair evaluation, we randomly picked one of the ten train-test splits. Then we trained an MLP classifier using the previously tuned hyperparameters on the train set. Once the training was done, we used two frameworks to evaluate our model. In the first framework, we used the entire independent test set to assess the performance of the trained MLP. This framework does not imitate real-life circumstances in which the percentage of typically developing students is higher than students with dyslexia. To tackle this issue, we fixed the number of typical readers and randomly chose three students with dyslexia; we repeated this process ten times and computed the average and standard deviation of the metrics. Table 13 shows the results.

**Table 13:** The comparison and validation of the MLP classifier on independent test data set.

Method	Metrics			
	Precision	Recall	*F*1-score	ROC-AUC
Previously obtained	0.913 ± 0.002	0.911 ± 0.002	0.912 ± 0.002	0.983 ± 0.000
Framework 1	0.859	0.812	0.800	0.786
Framework 2	0.878 ±0.112	0.883 ± 0.067	0.860 ± 0.095	0.767 ± 0.133

**Fig 5:**
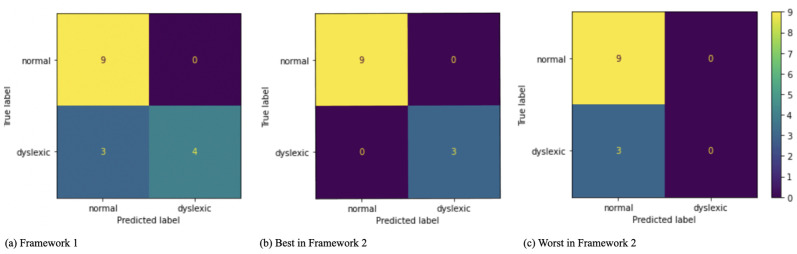
The confusion matrices of the MLP classifier on an independent test set using the combination of demographic and fixation: (a) the first framework results, (b) the best, and (c) the worst obtained results from the second framework.

Although we observed adequate performances in both of these two frameworks, in both of these frameworks, we observed some deteriorations between the independent test results and the previous results. These deteriorations became more evident between the ROC-AUC values. To explore the reason for such deteriorations, we scrutinized each of the individual predictions. Fig 5 demonstrates the confusion matrices of the first framework and the best results and the worst results of the second framework.

The algorithm mistakenly predicted three female students with dyslexia–from the first, second, and fourth grades–as typical readers. The reasons for such misclassifications could be: 1) due to the imbalanced representations of data, which becomes even more exaggerated in the first and second grades students’ data; 2) the lack of sufficient training data; 3) the occurrence of the so-called “distribution-shift”; 4) the lack of sufficiently informative features to enable the algorithm(s) to distinguish data points like these three samples from the corresponding typical readers.

To tackle the first shortcoming, we exploited various up/down-sampling techniques; to address the second shortcoming we applied data augmentation techniques. However, our results showed that none of these techniques solved the problem. Therefore, we concluded that collecting more data is key to tackling these two shortcomings as well as the third one.

To tackle the fourth issue, we see at least three ways to proceed (i) examining various feature combinations using the different data sources simultaneously to form a more functional feature space; (ii) adopting more complex methods like [60, 61]; (iii) narrowing down the problem into a smaller set of problems and adopting one-class classification methods like [62]. We postponed these items to our future studies.

## Conclusion and future work

The central objective of the current research was (i) to address the shortcomings of previously introduced data sets, (ii) to propose a robust AI-based solution to detect dyslexia at its early stages, and (iii) to investigate our psycholinguistic knowledge with the performance of our best AI model. To elaborate (i), the overwhelming majority of the previous data sets consisted of a small number of participants and the distributions of the control group and participants with dyslexia were synthetically balanced. More importantly, the age range of most of those data sets is inappropriate for correcting developmental dyslexia. Therefore, for the first time using the Russian language, we introduced a new eye movement data set consisting of 307 expert-annotated participant data, making it the largest data set in this category containing the most precise eye-tracking data. Not only does it mimic the real-world imbalanced data distributions of the normal and the dyslexic groups, but also it covers a broader and more appropriate age range (first- to sixth-grade primary-school students). Our data set consists of three sources of data 1) eye-fixation, 2) interest area, and 3) demographic, including the measure of IQ. We also introduced a new class by separating conventional dyslexia into those at low risk and those at high risk of dyslexia.

To achieve (ii), we investigated the performance of 12 classification approaches (from four families of models) on the individual subsets of our data set and their combination. In each of these cases, we fine-tuned the models using the BO method; after that, we trained and evaluated each model using a ten-fold cross-validation procedure and reported the average and standard deviation of the evaluation metrics. Our experiments showed that although no model obtained completely satisfactory results for detecting dyslexia from each of our single data sources, the CNN with *F*1-score = 0.637 and ROC AUC = 0.758 obtained the best, and relatively satisfactory, results for predicting dyslexia from the fixation data. And GB obtained nearly similar results on IA data. The combination of fixation and demographic data sources led four models to obtain acceptable results. Concretely, MLP with average *F*1-score = 0.912 and ROC AUC = 0.983 is our proposed AI model, while RF, GB, and KNN are also reliable alternatives. We observed more or less similar patterns and results on the combination of interest area with demographic data.

As for the advantages and disadvantages of the applied approaches, although our data set is the largest data set of its type, due to the limited training samples, we had to limit our experiment to shallower neural networks that are less prone to overfitting compared to deeper networks. Despite this fact, the neural networks in our experiments led to slightly better results than their competitors based on ensemble learning, interpreting their weights without exploiting tools like Shapley values is quite difficult—if not impossible—while interpreting and visualizing decision trees of limited size is far simpler.

In pursuit of our third objective, we exploited the SHAP approach to determine the importance of the features of one of our best classifiers on the fixation-demographic data set. In a nutshell, we observed that IQ, age, and being male are the top three (probably confounding) demographic features. Also, we observed the fixation along the *y*-axis is more important than the *x*-axis, the entire eye fixation data incorporates only 6.8% of the SHAP value’s share in identifying dyslexia. Our findings are partially aligned with our psycholinguistic domain knowledge.

In addition to the standard evaluation procedure, we assessed our best classifier’s performance on an independent test data set. Although the results of this test were acceptable, we observed some fluctuations in our evaluation metrics. Our investigation to figure out the reasons for these fluctuations led us to discover a three-year delay similarity between the eye movements of the first-grade typically developing students and the fourth-grade students with dyslexia.

The current study is not without limitations and shortcomings. Our data suffers from a lack of sufficient dyslexic samples for the first, second, and sixth grades. More samples are also needed for the newly proposed “at risk of dyslexia” class. Considering the fact that the demographic features are more important than fixation, thus the current performance of our proposed solution is conditioned on the demographic data, and as a matter of fact, we can not expect a very outstanding generalization power of AI model(s) for the grades lack of sufficient dyslexic samples or for the “at risk of dyslexia” class, unless we collect more data. Another shortcoming is that our data set is language-specific, although it expands the linguistic range of available datasets. Finally, our unreported experiments with clustering methods led to poor results; analyzing and improving those results is a matter for further study.

We see the following directions for our future study:

1. collecting more data covering the grades with small dyslexic samples and DR class,
2. launching several clinical trials of our proposed solution to assess the quality of the proposed solution rigorously and to collect more data;
3. adding new features or combining the existing ones as inputs to AI models so that we can deduce the importance of demographic data and increase the impact of eye-movement data,
4. applying more advanced classification methods, for instance, [60], or cluster analysis methods [63] by extending the concept of feature-rich networks to model the demographic-fixation data set, or applying reinforcement learning methods,
5. introducing a new data structure for handling the fixation data,
6. a deep investigation to determine whether the demographic features are confounders or colliders,
7. a deeper investigation to justify why the fixation along the y-axis is more important than the x-axis,
8. investigating the impact of demographic data and their combination with other publicly available eye-movement data sets from the literature.
